# Clinical trial to verify the safety of an automatic electric airway suction system: a multicenter prospective randomized study

**DOI:** 10.1186/s40001-024-02126-6

**Published:** 2024-11-29

**Authors:** Han Young Lee, Hoonsung Park, Seungmin Baik, Jae-myeong Lee

**Affiliations:** 1 Division of Acute Care Surgery, Department of Surgery, Korea University Anam Hospital, 73 Goryeodae-ro Seongbuk-gu, Seoul, 02841 Republic of Korea; 2 Division of Critical Care Medicine, Department of Surgery, Ewha Womans University Mokdong Hospital, Seoul, Republic of Korea

**Keywords:** Airway management, Suction drainage, Respiratory mucosa, Pneumonia, Ventilator-associated, Equipment and supplies, Treatment outcome

## Abstract

**Background:**

Acute lung injury and respiratory failure in patients in the intensive care unit (ICU) present challenges owing to varied factors. Manual suctioning, which is the current standard, burdens healthcare professionals and poses risks. The Lmeca.A1000 automatic suction device offers an innovative solution to this problem. This study aimed to evaluate the safety of this device.

**Methods:**

This prospective, multicenter, randomized trial compared automatic and manual suction in ICU patients. Mucosal injury and pneumonia were assessed using bronchoscopy and monitoring. Mechanical stability and adverse events were also evaluated.

**Results:**

In total, 117 critically ill adults were screened: 56 in the experimental group and 53 in the control group completing the study. No significant difference in mucosal injury was found at 3 or 7 days after treatment (*p* = 0.871, and 0.750). The incidence of VAP over 2 weeks was 5.36% in the experimental group and 1.89% in the control group, not significantly different (*p* = 0.582). Adverse events occurred in 14.3 and 13.2%, respectively, with no significant difference (*p* = 0.870). The incidence of clinical trial device malfunction within 3 days of application was 0.03% and within 2 weeks of follow-up was 0.02%.

**Conclusions:**

This study evidenced the non-inferiority of the automatic suction device to the manual method in terms of safety. The adoption of an automated system could alleviate the workload of healthcare professionals while maintaining effective airway management.

## Background

Acute lung injury and respiratory failure can arise from inflammatory reactions that damage lung epithelial cells and capillary structures owing to various causes [1]. With an increase in factors such as fine dust, contagious respiratory infections, aging populations, and conditions such as sepsis, multiple trauma, and stroke, the prevalence of acute lung injury and respiratory failure in intensive care unit (ICU) patients is significant. Literature reports suggest a prevalence rate of approximately 10–20% among ICU patients, with a high fatality rate [1–3]. Moreover, pneumonia is a significant contributing factor to lung damage, highlighting the importance of aspiration and removal of airway pollutants [4–6]. Consequently, the clearance of pollutants from the respiratory tract is increasingly critical, not only in ICU settings but also in general hospital wards, long-term care facilities, and home nursing care. Currently, airway management is performed manually at the patient’s bedside by healthcare professionals, mainly nurses and caregivers. The aspiration and removal of contaminants from the respiratory tract should only be performed when secretions are present [7]. However, when secretion volumes are substantial, the frequency of suctioning increases, leading to a higher workload for the nursing staff [8]. Failure to perform suction at appropriate intervals may result in issues such as increased oxygen demand. The appropriateness and safety of airway suctioning may vary depending on the proficiency of the individual performing it [9, 10]. Airway suctioning is an invasive procedure, and damage to the airway mucosa often occurs owing to the vacuum pressure generated by the suction catheter. In patients reliant on high-concentration oxygen, instances of hypoxia and cardiac arrest have been reported when the oxygen supply is disrupted during suctioning, or when suctioning is performed inconsistently in terms of strength. Furthermore, open-airway suctioning increases the risk of airborne and droplet transmission of infections to the surrounding areas, thereby increasing the potential for cross-infection. Simultaneously, there are concerns regarding medical expenses because disposable consumables must be discarded after each use [11–14].

Moreover, if a patient has a contagious respiratory illness, such as tuberculosis or coronavirus disease 2019 (COVID-19), healthcare personnel face an increased risk of infection when entering isolation rooms to conduct airway suctioning. In addition, each time a healthcare worker enters an isolation room, additional medical expenses are linked to changing clothing to prevent infections.

To enhance the existing manual airway suctioning process, Lmeca Co., Ltd. (Paju-si, Gyeonggi-do, South Korea) developed a medical device capable of unmanned automatic airway suctioning while adhering to the closed suction catheter method, with consultation of pulmonologists and anesthesiologists. The automatic suction device used in this study has been available in the domestic market since approval from the Korea Ministry of Food and Drug Safety. Notably, this medical device is not only the first of its kind in South Korea, but also has the potential to be a global pioneering innovation.

This study aimed to evaluate the effectiveness and safety of this medical device in real clinical settings and to assess any additional benefits it may offer during use.

## Methods

### Study design and patient selection

This study was designed as a prospective, multicenter, randomized, parallel, two-arm, non-inferiority trial with a 1:1 allocation ratio. Two adult surgical and two adult medical ICUs from three institutes are participate for the study. Patients admitted to the ICU of a clinical trial institution were divided into two groups: an experimental group that underwent automatic airway suction using a test device, and a control group that underwent manual airway suction performed by professional medical staff. The safety of these approaches were compared and evaluated. Owing to the inherent characteristics of the medical devices used in clinical trials, in which it is possible to ascertain whether a treatment has been administered to a subject based on the presence or absence of a mechanical device, this trial was conducted as an open-label study. In an open-label trial, the tester and the subject were aware of the group to which they have been assigned, eliminating the need for separate blinding.

Among patients admitted to the ICU, the investigator pre-screened adult patients aged 19 years or older who were undergoing mechanical ventilation and unable to expel sputum independently, relying on airway suction by medical staff. These patients were eligible for inclusion in the study. The investigator assigned sequential screening numbers only to participants who were expected to meet the selection criteria through pre-screening and who agreed to participate in the clinical trial. Subsequently, a screening test was conducted to confirm the suitability of participants. Following the test, only subjects deemed suitable for participation were assigned a randomization number and allocated to the appropriate treatment group. The screening and randomization numbers, along with the subjects’ initials, were used as subject identification codes.

Airway suction using a clinical trial medical device or closed suction catheter was administered to subjects enrolled in this clinical trial for a minimum of 3 days (72 h), with observations continuing for 2 weeks (14 days) from the commencement of the trial. Bronchoscopy and bronchial lavage tests were conducted to monitor mucosal injury, and sputum culture tests were performed immediately before the start of the trial and 3, 7, and 14 days later. The bronchoscopy procedure was documented and photographed, and each of the three intensive care physicians assessed the extent of injury to the airway mucosa using a five-grade scale (grade 0, normal; grade 1, erythema or edema; grade 2, erosion; grade 3, hemorrhage; and grade 4, ulceration or necrosis) [15].

We gathered demographic data (sex, date of birth, height/weight), vital signs, and medical histories of all patients participating in the clinical trial. In addition, pneumonia-related assessments (presence or absence of pneumonia and pneumonia severity score using the CURB-65 criteria), APACHE II (Acute Physiology And Chronic Health Evaluation II), SAPS III (Simplified Acute Physiology Score III), length of stay in the ICU, type of artificial airway used, mechanical ventilator settings, and recent sputum culture results were examined.

Throughout the follow-up period, chest X-rays, blood tests [white blood cell count (WBC), hemoglobin, hematocrit, platelet count, absolute neutrophil count (ANC), activated partial thromboplastin time (APTT), prothrombin time (PT), international normalized ratio (INR)], arterial blood gas analysis (ABGA), and inflammatory markers [procalcitonin, C-reactive protein (CRP)] were conducted. Bronchoscopy and bronchial lavage were performed to assess the grade of airway mucosal injury and to collect specimens for sputum culture.

All subjects were eligible to participate in the clinical trial only if they met the following criteria:

1. Patients aged 19 years or older admitted to the ICU of a clinical trial institution.
2. Patients whose duration of mechanical ventilation did not exceed 36 h.
3. Patients receiving mechanical ventilation via an artificial airway such as tracheal intubation or tracheostomy, with a ratio of partial pressure of oxygen in arterial blood (PaO_2_) to fraction of inspiratory oxygen concentration (FiO_2_) of 100 mmHg or higher.
4. Those expected to require continued mechanical ventilation for at least 72 h after the initiation of this clinical trial.

Subjects were excluded from this clinical trial if any of the following criteria applied:

1. Refusal to participate in the clinical trial by the subject or legal representative.
2. Pregnancy or plans to become pregnant during the trial participation period.
3. Patients with mechanical ventilator FiO_2_ exceeding 80% or plateau pressure exceeding 30 cmH_2_O.
4. Severe immune dysfunction, such as advanced blood cancer, bone marrow transplant failure, agranulocytosis, or ANC <500/mm^3.^
5. Individuals with unstable cardiovascular conditions such as hemodynamically unstable arrhythmia or severe hypoxemia.
6. Patients with ongoing bleeding owing to hemorrhagic causes such as multiple trauma or thrombocytopenia.
7. Patients scheduled for solid organ transplant surgery or whose spontaneous circulation was restored after cardiopulmonary resuscitation (CPR) on the day of trial participation.
8. Patients with lung disease accompanied by hemoptysis.

The clinical trial was terminated early if one or more of the following events occurred:

1. Withdrawal of consent to participate in the clinical trial by the subject or legal representative, or if the subject initially participated in the trial with the consent of the legal representative but later regained consciousness and declined to continue participation.
2. Concurrent treatment with surgery, medications, or other medical devices that could influence safety or effectiveness evaluation.
3. Complete suspension of medical device application for clinical trials to address adverse events.
4. Failure of the subject to adhere to treatment protocols.
5. Non-compliance with the provisions outlined in the consent form, affecting evaluation.
6. Death of the subject owing to an underlying disease unrelated to participation in the clinical trial.
7. Self-extubation occurring within 72 h after application of the clinical trial medical device to the subject.
8. Other instances where the investigator determined that there were issues affecting the progress of the clinical trial.

The clinical trial for the subject was discontinued if one or more of the following events occurred:

1. The clinical trial director requested that the Clinical Trial Review Committee terminate or suspend the clinical trial early, and the committee decided to stop the trial owing to difficulties observed during the trial, which rendered it impractical to continue.
2. The clinical trial sponsor requested that the Clinical Trial Review Committee terminate or suspend the clinical trial early, citing concerns regarding the safety of the clinical trial medical device, and the committee decided to halt the trial.
3. Temporary suspension of the trial occurred to address adverse events that occurred.
4. Discontinuation of the trial was warranted owing to the occurrence of a serious adverse event or adverse medical device reaction.

### Intervention and device information

**Fig. 1:**
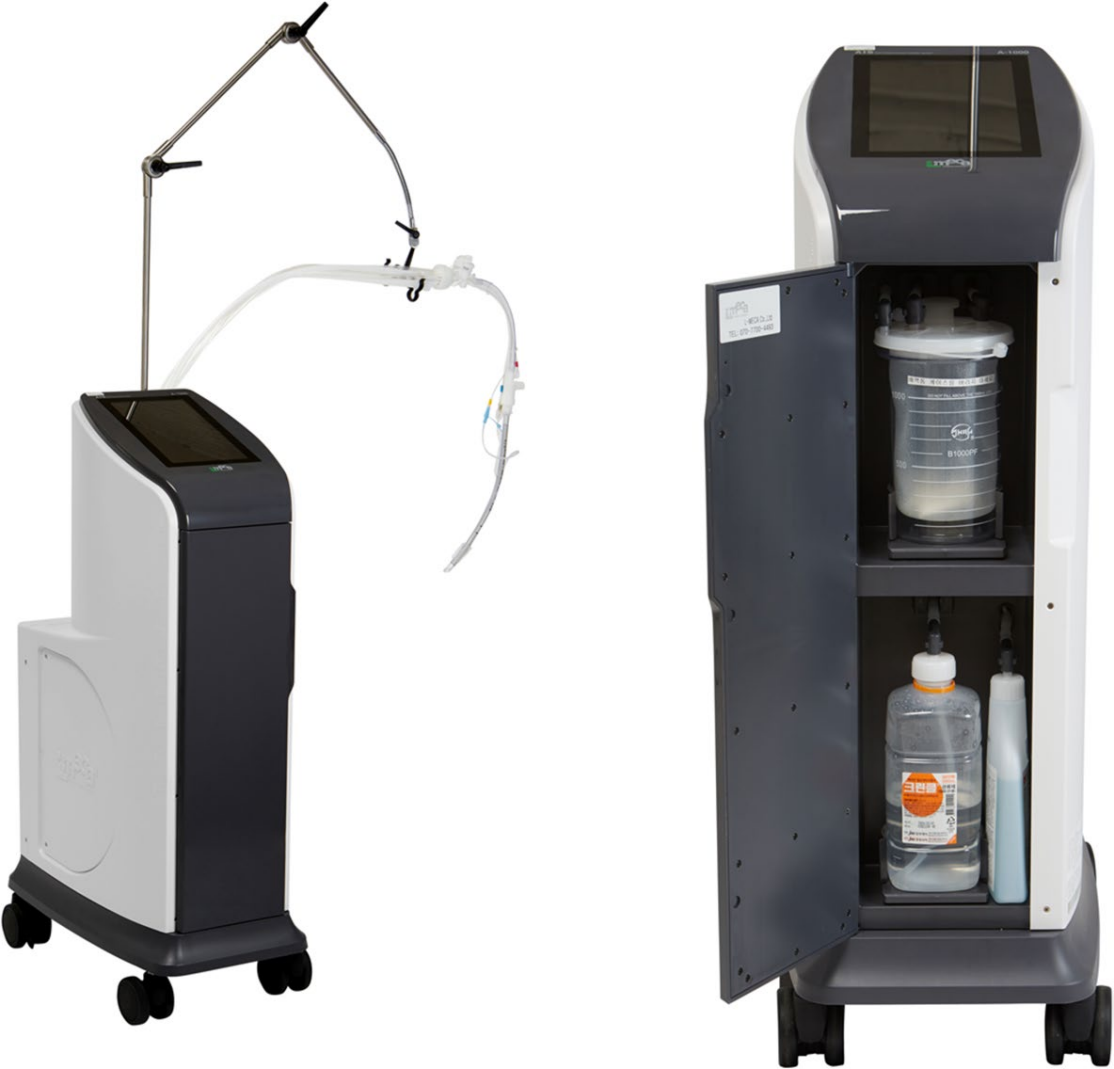
Novel automatic airway suction device (LMECA.A1000)

Airway sputum aspiration was performed in the experimental group by using an electric automatic sputum suction device. This clinical trial device (model name: A-1000, manufactured by Lmeca, Hwahap-ro, Bubwon-eup, Paju-si, Gyeonggi-do, South Korea) was designed to automatically remove airway secretions based on preset time intervals, pressure settings, and suction catheter insertion lengths (see Fig. 1). The device allows for customization by inputting the type and length of the airway tube as well as the desired advancement length of the suction catheter from the distal end of the airway tube, enabling individualized application for each patient. The suction catheter was designed as a closed system and was maintained for 24 h before replacement. It takes 10 s for each negative aspiration, and power of negative pressure was 180 mmHg.

In the control group, suctioning was performed by medical personnel using a closed suction catheter in a conventional manner.

In all cases, airway suction was performed at intervals ranging from 30 to 60 min.

### Outcomes

#### Primary outcome

Bronchoscopy was performed before and 3 days (72 h) after the application of the airway suction device. The test results were assessed by three clinicians who categorized the degree of injury to the airway mucosa resulting from airway suction into five grades to compare the differences between the two groups (grade 0, normal; grade 1, erythema or edema; grade 2, erosion; grade 3, hemorrhage; and grade 4, ulceration or necrosis).

#### Secondary outcome

Bronchoscopy was conducted before and 7 and 14 days after the application of the airway suction device. The test results were evaluated by three clinicians, who categorized the degree of damage to the airway mucosa resulting from airway suction into five grades to assess the differences between the two groups.

The occurrence of ventilator-associated pneumonia in the experimental and control groups was monitored for 2 weeks following the application of the airway suction device. Differences in the incidence rates between the two groups were compared. During the clinical trial period, chest radiography was conducted once every morning and more frequently, if necessary, up to twice a day. Ventilator-associated pneumonia was diagnosed if new pneumonia lesions appeared on the chest radiograph, along with two or more of the following conditions:

1. Patient body temperature exceeding 38 °C or <36 °C.
2. WBC count exceeding 12,000/mm^3^ or <4000/mm^3^.
3. Identification of bacteria through sputum culture or bronchoalveolar lavage culture test.
4. Discharge of purulent sputum through the lung bronchi.
5. ABGA results indicating decreased gas exchange or increased oxygen demand.

To assess the mechanical stability of the equipment, instances of malfunction were investigated within 72 h of application and during the follow-up period (2 weeks) in cases where the test device was applied to the experimental group.

#### Safety outcome

To assess the safety of the airway suction devices, we collected data on all adverse events during the follow-up period and investigated whether they were related to the use of airway suction devices.

### Sample size

Injury to the airway mucosa resulting from conventional passive airway suction practices has been reported to occur at varying frequencies. Erythema, edema, and erosions are commonly observed in patients with bronchitis or pneumonia. Significant airway mucosal injury owing to airway aspiration was defined as grade 3 or higher. According to a study reported in 2014, among 28 patients treated with a closed airway suction catheter for 48 h, the rate of airway mucosal injury of grade 3 or higher was observed in 38.7% of bronchoscopy results [15]. Another study involving 35 children reported that the frequency of moderate or severe airway injuries was 25.7% when using a closed airway suction catheter [16].

A pilot study conducted in 2019 focusing on the electric airway suction device used in this study revealed that 20% of the patients exhibited airway mucosal injury of grade 3 or higher on bronchoscopy. When comparing the degree of injury to the airway mucosa after application of the electric airway suction device for more than 48 h, no patient experienced an increase in grade by more than two levels. Considering the maximum incidence of mucosal injury (38.7%) when using a closed airway suction catheter and the maximum incidence of mucosal injury (20%) observed in the pilot study of an electric airway suction device, the non-inferiority limit was set at −20% in this study. To assess the non-inferiority of the electric airway suction device, more than 140 subjects were required, assuming a minimum power of 80%, dropout rate of 10%, and one-sided significance level of 2.5%. The sample size for this clinical trial was calculated using the "Non-Inferiority Tests for the Difference Between Two Proportions" procedure in PASS 2020 (Power Analysis and Sample Size Software, NCSS, LLC, Kaysville, Utah, USA).

### Randomization

After screening, all the study participants were enrolled based on the principal investigator’s decision at each institution. The randomization ratio between the test and control groups was 1:1 and a permuted block randomization method was employed at each clinical trial institution. The randomization code was generated using SAS version 9.4 (SAS Institute Inc., Cary, North Carolina 27513, USA). The block size and seed number were randomly selected by the personnel responsible for randomization at each institution. The randomization number was created by combining a one-digit number representing each institution, “R” for random assignment, and a three-digit number representing the order of subjects. Following randomization, each intervention was administered by an assigned nurse for the study subject.

### Statistical analysis

Statistical analyses were performed using SAS version 9.4 (SAS Institute Inc., Cary, North Carolina, USA). Continuous variables were expressed as mean ± standard deviation (SD) or median with interquartile range (IQR), whereas categorical variables were presented as numbers and percentages. All statistical tests in the clinical trials were two-sided with a significance level of 0.05, and the analysis included intra- and inter-group comparisons.

#### Primary outcome

Before application and 3 days (72 h) after applying the airway suction device, the number and proportion of participants with grade 3 or higher airway mucosal injury at each time point were presented by group. Statistical significance of the difference in airway mucosal injury between the experimental and control groups was tested using the chi-squared test or Fisher’s exact test. If the 95% confidence interval exceeded −20%, it was considered successful, satisfying non-inferiority.

#### Secondary outcome

Before application and at 7 and 14 days after the application of the airway suction device, the number and proportion of participants with airway mucosal injury of grade 3 or higher at each time point were presented by group. The statistical significance of the difference in airway mucosal injury between the experimental and control groups was evaluated using the chi-squared test or Fisher’s exact test for comparison assessment.

Regarding the incidence of ventilator-associated pneumonia in the test and control groups evaluated by the investigator for 2 weeks of the clinical trial, the number and proportion of subjects who developed pneumonia were presented by group. The statistical significance of the difference in pneumonia incidence between the test group and the control group was tested using the chi-squared test or Fisher’s exact test. If the 95% lower confidence limit was greater than −20%, non-inferiority was satisfied, and it was judged to be successful.

The test device applied to the test group was considered valid if it operated for more than 90% of the time during the 3-day (72 h) and 14-day follow-up period without any device malfunction.

#### Safety outcome

The safety of all subjects enrolled in this study and receiving the clinical trial medical devices was evaluated. Subjects for whom safety information was not collected at follow-up were excluded from the analysis. The severity, causality, number of adverse events, and frequency and percentage of occurrence are presented for all reported adverse events. For continuous variables, the mean, SD, median, minimum, and maximum values were presented, and for categorical data, frequencies and ratios were presented. Continuous variables were compared using an independent two-sample *t* test or the Mann–Whitney *U* test, depending on the normality of each group. Categorical variables were compared using the Pearson’s chi-squared test or Fisher’s exact test.

## Results

**Fig. 2:**
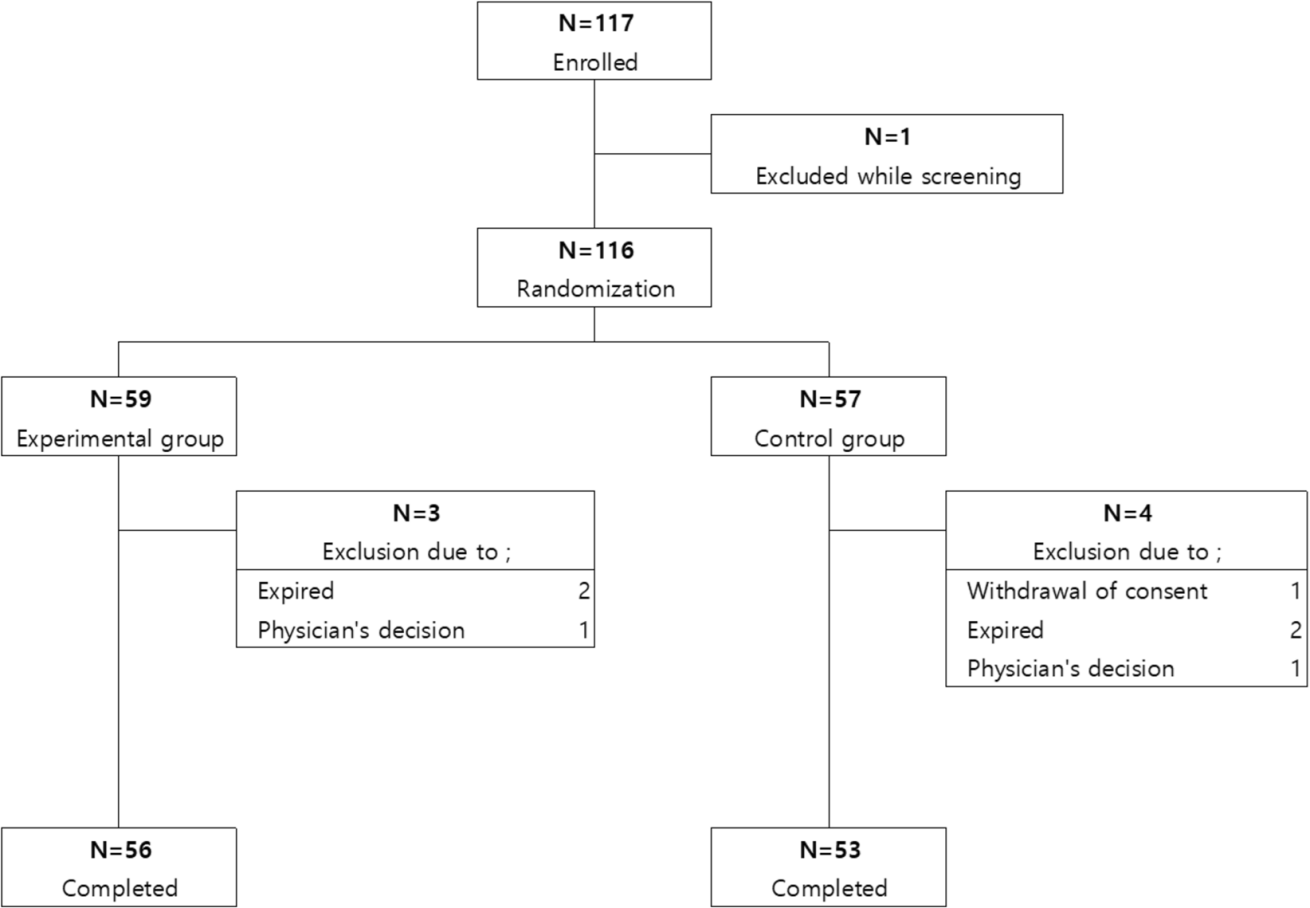
Flowchart of this study

Between March 2021 and July 2023, 117 critically ill adults were screened across three institutions. Following the primary exclusion criteria and randomization, 59 individuals were enrolled in the experimental group and 57 were enrolled in the control group. Ultimately, 56 patients from the experimental group and 53 from the control group completed the study, with some exclusions occurring during the study period (Fig. 2).

The average age of the 109 subjects who participated in the clinical trial was 67.4 ± 16.1 years, with 73 (67.0%) men and 36 (33.0%) women, and an average height of 163.2 ± 10.4 cm. No statistically significant differences were observed between the test and control groups in terms of age, sex, or height. The average weight of the 56 individuals in the test group was 68.1 ± 18.6 kg, whereas the average weight of the 53 individuals in the control group was 59.5 ± 12.6 kg, indicating a statistically significant difference between the test group and the control group (*p* value = 0.0135) (Table 1).

**Table 1:** Comparison of clinical demographics and characteristics at time of study enrollment

	Experimental group *N* = 56 *n* (%)	Control group *N* = 53 *n* (%)	Total *N* = 109 *n* (%)	*p* value^1^
Age (years)				
Mean ± SD	67.5 ± 16.6	67.3 ± 15.7	67.4 ± 16.1	0.7942^b^
Range	(22–88)	(26–90)	(22–90)	
Sex				
Male	38 (67.8)	35 (66.0)	73 (67.0)	0.8400^e^
Female	18 (32.2)	18 (34.0)	36 (33.0)	
Height (cm)				
Mean ± SD	164.4 ± 10.1	161.9 ± 10.6	163.2 ± 10.4	0.2706^b^
Range	(140.0–184.0)	(128.5–180.0)	(128.5–184.0)	
Weight (kg)				
Mean ± SD	68.1 ± 18.6	59.4 ± 12.6	63.9 ± 16.5	**0.0135**^**b**^
Range	(34.3–132.2)	(34.3–90.0)	(34.3–132.2)	
APACHE II				
Mean ± SD	27.11 ± 10.00	28.17 ± 7.60	27.62 ± 8.89	0.5321^a^
Range	(6–45)	(13–51)	(6–51)	
SAPS III				
Mean ± SD	69.1 ± 19.5	70.8 ± 14.8	69.9 ± 17.3	0.6034^a^
Range	(34–124)	(34–97)	(34–124)	
ICU length of stay (days)				
Mean ± SD	3.8 ± 5.6	2.7 ± 3.6	3.3 ± 4.7	0.1490^b^
Range	(0–36)	(0–17)	(0–36)	
Tube type				
E-tube	54 (96.4)	50 (94.3)	104 (95.4)	0.6730^f^
T-tube	2 (3.6)	3 (5.7)	5 (4.6)	
Tube diameter (cm)				
Mean ± SD	7.58 ± 0.34	7.50 ± 0.31	7.54 ± 0.33	0.2060^b^
Range	(7.0–8.0)	(6.5–8.0)	(6.5–8.0)	
PaO_2_/FiO_2_ (mmHg)				
Mean ± SD	275.2 ± 151.0	285.1 ± 139.2	280.0 ± 144.8	0.4990^b^
Range	(102–799)	(102–675)	(102–799)	
Underlying pneumonia				
Yes	30 (53.6)	28 (52.8)	58 (53.2)	0.9382^e^
No	26 (46.4)	25 (47.2)	51 (46.8)	
CURB-65 (Pneumonia severity score)				
	*n* = 30^2^	*n* = 28^2^	*n* = 58^2^	
Mean ± SD	2.7 ± 1.0	2.8 ± 1.2	2.8 ± 1.1	0.7215^b^
Range	(0–4)	(0–4)	(0–4)	
Sputum culture (Gram stain results)				
Positive	26 (46.4)	26 (49.1)	52 (47.7)	0.7837^e^
Negative	30 (53.6)	27 (50.9)	57 (52.3)	
WBC (×10^3^ µL)				
Mean ± SD	13.0 ± 9.8	11.1 ± 7.5	12.1 ± 8.8	0.3489^b^
Range	(0.3–46.2)	(1.0–40.8)	(0.3–46.2)	
ANC (/µL)				
Mean ± SD	11,272.5 ± 9220.4	9492.9 ± 6692.9	10,407.2 ± 8103.2	0.4288^b^
Range	(140.0–40608.0)	(619.0–37037.0)	(140.0–40608.0)	
PT (INR)				
Mean ± SD	1.5 ± 0.8	1.5 ± 0.4	1.5 ± 0.7	0.3069^b^
Range	(0.9–6.5)	(0.5–3.2)	(0.5–6.5)	
CRP (mg/L)				
Mean ± SD	99.2 ± 76.3	95.1 ± 77.5	97.2 ± 76.6	0.7920^b^
Range	(0.3–288.2)	(0.2–313.6)	(0.2–313.6)	
	*n* = 54^3^	*n* = 52^3^	*n* = 106^3^	
Procalcitonin (ng/mL)				
Mean ± SD	19.4 ± 32.8	20.5 ± 36.8	19.9 ± 34.7	0.5357^b^
Range	(0.0–119.8)	(0.1–194.6)	(0.0–194.6)	

*SD* standard deviation, *ICU* intensive care unit

Continuous values were written as mean ± SD (range)

Categorical values were written as number (percentage)

^1^a: Independent two-sample *t* test, b: Wilcoxon’s rank-sum test, e: chi-squared test, f: Fisher’s exact test, value lower than 0.05 is marked as bold text

^2^Only among patients with present pneumonia

^3^Sample losses due to missing lab results (1 from control, 2 from experimental group)

The average APACHE II score was 27.6 ± 8.9, the SAPS III score was 69.9 ± 17.3, and the average length of stay in the ICU was 3.3 ± 4.7 days. There were no statistically significant differences between the two groups (Table 1).

Upon checking the vital signs of the 109 subjects just before the start of the trial, there was no statistically significant difference between the test and control groups in terms of systolic blood pressure, diastolic blood pressure, pulse rate, body temperature, and respiratory rate (Table 1).

Regarding the airway tube and mechanical ventilator settings, 104 subjects (95.4%) used an endotracheal tube, whereas 5 subjects (4.6%) used a tracheal tube. There were no significant differences between the two groups in the average internal diameter of the airway tube used and mechanical ventilator parameters before the application of the medical device (peak pressure, plateau pressure, expiratory tidal volume, and FiO_2_). However, there was a significant difference in the positive end expiratory pressure (PEEP) before application of the medical device, which was 8.4 ± 2.9 cmH_2_O in the test group and 7.3 ± 2.3 cmH_2_O in the control group (*p* value = 0.0488).

Of the 109 patients, 58 (53.2%) were confirmed to have pneumonia at the start of the study. The mean pneumonia severity score (CURB-65) was 2.8 ± 1.1 points. Prior to using the medical device, sputum tests (Gram stain) revealed that 52 subjects (47.7%) had pathogenic bacteria instead of normal flora, whereas 57 subjects (52.3%) did not. There were no statistically significant differences between the two groups in terms of the presence of pneumonia, pneumonia severity score, or pathogenic bacteria (Table 1).

According to the ABGA results immediately before the start of the trial, there were no significant differences in PaO_2_, PaCO_2_, HCO_3_-, and pH between the two groups, and there was no difference in the PaO_2_/FiO_2_ ratio. In addition, there was no statistically significant difference between the test and control groups in WBC, ANC, PT, INR, CRP, and procalcitonin performed at the screening stage for 109 subjects (Table 1).

When examining past medical history that may influence systemic inflammation and airway mucosal injury, infections and infestations excluding pneumonia were found in 68 of 109 (62.4%) patients, pneumonia in 55 patients (50.5%), hypertension in 25 patients (22.9%), peritonitis in 17 patients (15.6%), and diabetes mellitus in 19 patients (17.4%). There was no statistically significant difference in medical history between the two groups.

### Primary outcome

There was no statistically significant difference between the two groups in the degree of injury to the airway mucosa before or 3 days (72 h) after application of the airway suction device. After 3 days (72 h) of application, grade 3 or higher injuries were observed in 6.1% (3 of 49) of the test group and 2.3% (1 of 43) of the control group. The 95% two-sided confidence interval for the difference between the test group and the control group had a lower limit of −0.04 and an upper limit of 0.12. The lower limit exceeded the non-inferiority limit (−0.2) (Table 2).

**Table 2:** Airway mucosal injury grade at initial enrollment, after 3 days and after 7 days

	Baseline		After 3 days		After 7 days	
Airway mucosal injury grade	Experimental group	Control group	Experimental group	Control group	Experimental group	Control group
	*n* = 56	*n* = 53	*n* = 49	*n* = 43	*n* = 18	*n* = 14
0 (normal)	23 (41.1)	15 (28.3)	12 (24.5)	10 (23.3)	5 (27.8)	4 (28.6)
1 (erythema or edema)	27 (48.2)	30 (56.6)	29 (59.2)	25 (58.1)	10 (55.5)	6 (42.9)
2 (erosion)	6 (10.7)	8 (15.1)	5 (10.2)	7 (16.3)	3 (16.7)	3 (21.4)
3 (hemorrhage)	0 (0.0)	0 (0.0)	2 (4.1)	1 (2.3)	0 (0.0)	1 (7.1)
4 (ulceration or necrosis)	0 (0.0)	0 (0.0)	1 (2.0)	0 (0.0)	0 (0.0)	0 (0.0)
*p* value^1^	0.3594^a^		0.8712^b^		0.7497^b^	
95% CI of mean difference			−0.04, 0.12		−0.19, 0.05	

*CI* confidence interval

^1^a: chi-squared test, b: Fisher’s exact test

### Secondary outcome

There was no statistically significant difference between the two groups in the degree of airway mucosal injury before, and 7 and 14 days after the application of the airway suction device. After 7 days of application, grade 3 or higher injuries were observed in 0% (0 out of 18) of the test group and 7.1% (1 out of 14) of the control group. The 95% two-sided confidence interval for the difference between the test group and the control group had a lower limit of −0.19 and an upper limit of 0.05. The lower limit exceeded the non-inferiority limit (−0.2). Only one patient in the experimental group remained in the trial until the 14th day, and there was no grade 3 or higher mucosal injury (Table 2).

The incidence of ventilator-associated pneumonia for 2 weeks after the application of the airway suction device was 5.36% (3.56) in the test group and 1.89% (1.53) in the control group. There were no significant differences between the two groups. The 95% two-sided confidence interval for the difference between the test group and the control group had a lower limit of −0.03 and an upper limit of 0.10. The lower limit exceeded the non-inferiority limit (−0.2).

The incidence of clinical trial device malfunction within 3 days (72 h) of application was 0.03% (2 out of 6100), and within 2 weeks of follow-up was 0.02% (2 out of 8297). More than 90% of the patients operated normally without malfunction during the 3-day and 2-week follow-up periods.

### Subgroup analysis

Upon examining the degree of injury to the airway mucosa before and 3 days (72 h) after the application of the airway suction device in the subgroup of subjects with a pneumonia severity score of <2 points at the beginning of the trial, no statistically significant difference was observed between the test and control groups. Similarly, in the subgroup of subjects with a pneumonia severity score of 2 or more at the beginning of the trial, there was no difference between the test group and the control group.

Furthermore, there was no statistically significant difference in the degree of injury to the airway mucosa in each analysis result when divided into male–female groups and groups aged <70 years and ≥70 years.

### Safety outcome

In this clinical trial, treatment-emergent adverse events (TEAE) occurred in 8 of 56 patients (14.3%) in the test group and in 7 of 53 patients (13.2%) in the control group. Among these, ventilator-associated pneumonia was reported in 3 cases (5.4%) in the test group and 1 case (1.9%) in the control group. Other notable adverse events include hypoxia, cardiac failure, bradycardia, tachycardia, and cardiac arrest. There was no statistically significant difference between the two groups in terms of adverse events. Hypoxia was confirmed in one patient (1.8%) in the test group, whereas cardiac disorders occurred in four patients (7.2%) in the test group and one patient (1.9%) in the control group. In the control group, hematuria and acute kidney injury were confirmed in five patients (9.5%). Cardiac arrest and bradycardia were confirmed in one patient in the test group; however, following a comprehensive review, it was determined that these events were not related to the clinical trial device. See Table 3 for details.

**Table 3:** Safety outcomes

Type	Experimental group	Control group	Total	*p* value^1^
	*N* = 56 *n* (%)	*N* = 53 *n* (%)	*N* = 109 *n* (%)	
Total number of treatment emergent adverse events	8 (14.3)	7 (13.2)	15 (13.8)	0.8703^e^
VAP diagnosed within study period	3 (5.4)	1 (1.9)	4 (3.7)	0.5820^f^
	95% CI of mean difference		−0.03, 0.10	
Hypoxia	1 (1.8)	0 (0.0)	1 (0.9)	1.0000^f^
Cardiac disorders				
Cardiac failure	2 (3.6)	0 (0.0)	2 (1.8)	0.4958^f^
Bradycardia	1 (1.8)	0 (0.0)	1 (0.9)	1.0000^f^
Tachycardia	0 (0.0)	1 (1.9)	1 (0.9)	1.0000^f^
Cardiac arrest	1 (1.8)	0 (0.0)	1 (0.9)	1.0000^f^

*VAP* ventilator-associated pneumonia

^1^e: chi-squared test, f: Fisher’s exact test

Regarding the vital sign measurements conducted during the clinical trial, there were no statistically significant differences between the groups in vital signs other than body temperature. Although there was no significant difference in body temperature between the two groups at the start of the trial, a notable difference was observed in the change in body temperature 3 days (72 h) after application of the airway suction device. Specifically, the body temperature in the test group exhibited a significant decrease of −0.2 ± 0.8 ℃, compared to the control group, which experienced an increase of 0.3 ± 0.9 ℃ (*p* value = 0.0058). Refer to Table 4 for detailed findings.

**Table 4:** Change of clinical features during study period

	Experimental group *N* = 56 *n* (%)	Control group *N* = 53 *n* (%)	Total *N* = 109 *n* (%)	*p* value^1^
BT (℃)				
Baseline				
* n*	56	53	109	
Mean ± SD	37.0 ± 0.8	36.6 ± 0.9	36.8 ± 0.9	0.0621^b^
Range	(36.0–40.0)	(33.0–38.0)	(33.0–40.0)	
Change from baseline after 3 days				
*n*	49	44	93	
Mean ± SD	−0.2 ± 0.8	0.3 ± 0.9	0.00 ± 0.9	**0.0058**^**b**^
Range	(−3.0 to 1.3)	(−1.6 to 3.0)	(−3.0 to 3.0)	
PaCO_2_ (mmHg)				
Baseline				
*n*	56	53	109	
Mean ± SD	37.3 ± 11.0	35.4 ± 10.8	36.4 ± 10.9	0.4558^b^
Range	(20.4–88.1)	(16.6–80.0)	(16.6–88.1)	
After 8 days				
*n*	7	9	16	
Mean ± SD	40.9 ± 7.4	33.5 ± 9.8	36.7 ± 9.4	**0.0343**^**b**^
Range	(33.5–54.1)	(20.8–56.6)	(20.8–56.6)	
Change from baseline after 8 days				
*n*	7	9	16	
Mean ± SD	7.8 ± 6.3	−6.5 ± 13.3	−0.2 ± 12.8	**0.0211**^**a**^
Range	(2.8–20.6)	(−30.5 to 8.1)	(−30.5 to 20.6)	
WBC (×10^3^ µL)				
Baseline				
*n*	56	53	109	
Mean ± SD	13.0 ± 9.8	11.1 ± 7.5	12.1 ± 8.8	0.3489^b^
Range	(0.3–46.2)	(1.0–40.8)	(0.3–46.2)	
After 8 days				
*n*	4	2	6	
Mean ± SD	8.4 ± 1.5	14.2 ± 3.4	10.3 ± 3.5	**0.0364**^**a**^
Range	(7.1–10.5)	(11.7–16.6)	(7.1–16.6)	
ANC (µL)				
Baseline				
*n*	56	53	109	
Mean ± SD	11,272.5 ± 9220.5	9492.9 ± 6692.9	10,407.2 ± 8103.2	0.4288^b^
Range	(140.0–40608.0)	(619.0–37037.0)	(140.0–40608.0)	
After 11 days				
*n*	4	2	6	
Mean ± SD	6396.0 ± 1039.2	12,207.0 ± 2996.7	8333.0 ± 3383.6	**0.0185**^**a**^
Range	(5148.0–7686.0)	(10,088.0–14326.0)	(5148.0–14326.0)	
PT (INR)				
Baseline				
*n*	56	53	109	
Mean ± SD	1.5 ± 0.8	1.5 ± 0.4	1.5 ± 0.7	0.3069^b^
Range	(0.9–6.5)	(0.5–3.2)	(0.5–6.5)	
Change from baseline after 2 days				
*n*	51	46	97	
Mean ± SD	0.0 ± 0.4	0.0 ± 0.4	0.0 ± 0.4	**0.0349**^**b**^
Range	(−1.6 to 2.1)	(−0.64 to 1.8)	(−1.6 to 2.1)	
Change from baseline after 3 days				
*n*	49	44	93	
Mean ± SD	0.0 ± 0.4	−0.1 ± 0.3	−0.1 ± 0.4	**0.0363**^**b**^
Range	(−1.5 to 1.1)	(−1.0 to 1.1)	(−1.5 to 1.1)	
Change from baseline after 4 days				
*n*	29	27	56	
Mean ± SD	0.0 ± 0.5	−0.1 ± 0.5	−0.1 ± 0.5	**0.0472**^**b**^
Range	(−1.6 to 1.5)	(−1.1 to 1.3)	(−1.6 to 1.5)	
Change from baseline after 5 days				
*n*	22	21	43	
Mean ± SD	−0.1 ± 0.4	−0.2 ± 0.4	−0.2 ± 0.4	**0.0400**^**b**^
Range	(−1.6 to 0.6)	(−1.1 to 0.7)	(−1.6 to 0.7)	
Change from baseline after 6 days				
*n*	20	16	36	
Mean ± SD	−0.2 ± 0.4	−0.2 ± 0.6	−0.2 ± 0.5	**0.0465**^**b**^
Range	(−1.7 to 0.3)	(−0.9 to 1.7)	(−1.7 to 1.7)	
Change from baseline after 7 days				
*n*	19	14	33	
Mean ± SD	−0.1 ± 0.5	−0.3 ± 0.4	−0.2 ± 0.4	**0.0146**^**b**^
Range	(−1.7 to 0.7)	(−0.7 to 0.8)	(−1.7 to 0.8)	
CRP (mg/L)				
Baseline				
*n*	56	53	109	
Mean ± SD	99.2 ± 76.3	95.1 ± 77.5	97.2 ± 76.6	0.7920^b^
Range	(0.3–288.2)	(0.2–313.6)	(0.2–313.6)	
Change from baseline after 10 days				
*n*	4	5	9	
Mean ± SD	−21.6 ± 6.9	53.2 ± 82.6	19.9 ± 70.6	**0.0200**^**b**^
Range	(−30.9 to −16.0)	(−15.4 to 155.8)	(−30.9 to 155.8)	

^1^a: Independent two-sample *t* test, values lower than 0.05 are marked as bold text

Laboratory test results revealed statistically significant differences in PaCO_2_, WBC, ANC, PT (INR), and CRP levels between the test and control groups. Although there was no initial discrepancy in PaCO_2_ levels between the two groups, the test group exhibited a higher value compared to the control group 8 days after application of the airway suction device (test group: 40.9 ± 7.4, control group: 33.5 ± 9.8, *p* value = 0.0343). In addition, there was a significant disparity in the degree of change on day 8 compared to the pre-application results (test group: 7.8 ± 6.3, control group: −6.5 ± 13.3, *p* value = 0.0211). WBC counts were higher in the experimental group before the start of the test, although the difference was not statistically significant. However, the results obtained 11 days after application of the airway suction device showed 8.4 ± 1.5 × 10^3^ µL in the test group and 14.2 ± 3.4 × 10^3^ µL in the control group, indicating a statistically significant decrease in the test group (*p* value = 0.0364). The ANC levels were also higher in the experimental group before the start of the test, although the difference was not significant. However, at 11 days after application of the airway suction device, the test group had 6396 ± 1039 µL and the control group had 12,207.0 ± 2996.7 µL, which was significantly lower in the test group (*p* value = 0.0185). The PT (INR) did not differ between the two groups before the start of the test. However, when measured 2 days (48 h), 3 days (72 h), 4 days, 5 days, 6 days, and 7 days after applying the airway suction device, the decrease was significantly greater in the control group (*p* value = 0.0349, 0.0363, 0.0472, 0.0400, 0.0465, and 0.0146, respectively).

There was no difference in CRP levels between the two groups before the start of the test. However, the level of change 10 days after applying the airway suction device showed a statistically significant decrease in the experimental group, with values of −21.6 ± 6.9 mg/L in the test group and 53.2 ± 82.6 mg/L in the control group (*p* value = 0.0200) (Table 4).

## Discussion

This study followed the ISO 14155:2020 guidelines for clinical trials. Patients undergoing mechanical ventilation through an artificial airway were divided into two groups: one using a test device (Lmeca.A1000, an electric medical suction device) and the other using a standard manual airway suction procedure. This study aimed to compare and assess the effectiveness and safety of the test device. To our knowledge, this multicenter, prospective, randomized, open-label, pivotal study is the first attempt to evaluate the safety of any automatic airway suction device.

For the primary assessment, the presence of grade 3 or higher airway mucosal injury was evaluated before and 3 days (72 h) after the application of the clinical trial medical device. No significant differences were observed between the two groups. The 95% two-sided confidence interval for the difference between the test group and the control group had a lower limit of −0.04 and an upper limit of 0.12. Notably, the lower limit exceeded the non-inferiority threshold (−0.2), indicating that the test group was not inferior to the control group, and thus met the criteria for non-inferiority.

For the secondary assessment, the presence of grade 3 or higher airway mucosal injury was examined before and 7 and 14 days after the application of the clinical trial medical device. However, on the 14th day, only one patient from the experimental group remained; thus, this patient was excluded from the analysis. There was no statistically significant difference in the degree of airway mucosa injury 7 days after application. The 95% two-sided confidence interval for the difference between the test group and the control group had a lower limit of −0.19 and an upper limit of 0.05. Remarkably, the lower limit exceeded the non-inferiority threshold (−0.2), indicating that the test group was not inferior to the control group and met the criteria for non-inferiority.

Furthermore, there was no statistically significant difference in the incidence of ventilator-associated pneumonia between the two groups throughout the 2 weeks following the application of the clinical trial medical device. The 95% two-sided confidence interval for the difference between the test group and the control group had a lower limit of −0.03 and an upper limit of 0.10. Notably, the lower limit exceeded the non-inferiority threshold (−0.2), indicating that the test group was not inferior to the control group.

The incidence of malfunction in the clinical trial medical device within 3 days (72 h) of application was 0.03%, and within 2 weeks of follow-up, it was 0.02%. These results indicate that the device operated effectively, with more than 90% of the patients functioning properly during the 3-day (72-h) and 2-week follow-up periods.

Based on these results, we concluded that the degree of injury to the airway mucosa caused by the use of the clinical trial medical device was not inferior to that caused by the previous manual method. In addition, the device operated normally more than 90% of the time, thereby demonstrating its effectiveness.

In this clinical trial, adverse events occurred in eight of 56 patients (14.29%) in the test group and seven of 53 patients (13.21%) in the control group. However, it was confirmed that there was no causal relationship with the use of the airway suction device. Cardiac arrest and bradycardia were confirmed in one patient each in the test group; however, both cases occurred during the resting period when the medical device did not perform suction. The subject who developed cardiac arrest of unknown cause had spontaneous circulation restored after two cycles of CPR and recovered after concurrent treatment with therapeutic drugs. Based on this, it was concluded that there was no difference in stability compared with the previous manual method.

An analysis was conducted to determine whether the use of clinical trial medical devices would improve vital signs, assist in gas exchange, and improve blood test indicators of inflammation. Statistically significant differences between the test and control groups were confirmed in temperature, WBC count, ANC, PT (INR), and CRP levels at some time points. However, this trend was not clear upon detailed analysis.

Compared to the start of the test, the degree of decrease in body temperature was significantly greater in the experimental group than in the control group after 3 days (72 h) of application. However, the average body temperature in both groups was within the normal range, and there were no significant differences between the two groups on other days. Other vital sign indicators did not show clear trends or statistically significant differences; therefore, the use of clinical trial medical devices could not be considered to have an effect on the improvement of vital signs.

ABGA revealed that the only significant difference between the two groups was PaCO_2_. On the 8th day of application of the airway suction device, PaCO_2_ was significantly higher in the experimental group than in the control group, and the degree of increase was significantly greater than that at the start of the test. However, there were no significant differences on other days, and there was no consistent trend of higher values in the experimental group. Despite the differences between the two groups, the average PaCO_2_ values in each group were within the normal range. The pH and bicarbonate levels were within the normal range for both groups at all time points, and there was no significant difference in PaO_2_ and PaO_2_/FiO_2_ ratio between the two groups. Based on these results, there was no evidence that the clinical trial medical device helped with gas exchange.

WBC and ANC levels were initially higher in the experimental group but tended to be consistently lower than those in the control group after 2 days (48 h). However, there was only 1 day when both indicators were significantly lower in the experimental group, and at that point, the sample sizes for both groups were very small (<5 patients). There was no consistent trend for CRP levels to be superior or inferior between the two groups, and there was a time when there was a statistically significant decrease in the experimental group compared with the start of the test; however, at this time, the number of patients in the two groups was ≤5. Procalcitonin levels tended to be consistently lower in the experimental group from day 6; however, there was no statistically significant difference between the two groups. In the case of PT (INR), the degree of decrease in PT (INR) in the control group was consistently greater during the observation period than that at the start of the test; however, there was no significant difference in the value of PT (INR) compared with the test group. Clinically, there were no differences in bleeding tendencies between the two groups. When these blood test results were combined, there was no evidence to suggest that the inflammatory response was further improved in the test group.

Using the device has potential to reduce the need for direct airway suction by the nurse, saving time by only needing to ensure that the machine’s settings were appropriate and deciding whether the application interval needed adjustment. Previously, missing the right time for airway suction owing to other tasks was a problem. However, automated devices enabled the maintenance of regular suction intervals, which many nurses appreciated for reducing their workload. However, the inability to objectively judge proper suction resulted in intermittent manual suction, potentially undermining the trust in the device among medical staff. Other concerns include doubts regarding device reliability. It was noted that the machine operates based on set intervals and times regardless of the patient’s reactions or abnormal signs, making it unable to address adverse reactions unless attended to by medical staff. Currently, the device lacks the capability to detect such changes and requires medical staff to recognize and intervene with alarms using patient monitors or mechanical ventilators. Future plans include establishing safety protocols to recognize excessive pressure changes in the airway or halt machine operations based on patient-monitoring readings.

### Limitations

The major limitation of this study was the inconsistency in the follow-up periods of all patients. Consequently, 6 days after initiating the observation period, only half of the participants in each group remained, compared with the beginning. This discrepancy may have introduced a bias in the analysis of the long-term follow-up period after 3 days. Particularly concerning the blood test results, significant differences between the two groups occurred when the number of patients in each group was reduced to 10 or fewer, rendering a meaningful analysis impractical.

Another potential limitation is the variability in physicians performing bronchoscopies across different institutions. However, post-study endoscopic findings indicated that the degree of damage for most subjects was consistently the same when evaluated independently by researchers at each institution in a double-blind manner. In instances of discordant opinions, a double-blind evaluation method was employed to reconcile the differences. Therefore, we believe that issues stemming from different observers were minimal.

Unlike in the experimental group, in the control group, airway suction was directly performed by the attending nurse, leading to potential variations in suction strength and application time depending on the individual medical personnel conducting the procedure. The standardization of these factors proved challenging, and only the application interval could be regulated uniformly. Consequently, we were limited in our ability to achieve consistent treatment across the control group.

This study did not assess the efficacy of foreign substance removal from the airways owing to the absence of a quantitative method to evaluate the effectiveness of airway suction in removing sputum. As the quantity of sputum present in the respiratory tract varies among patients, it is impractical to determine the extent of removal solely by measuring the aspirated sputum volume. When considering the measurement of aspirate collected in the drainage container as an indirect approach, it was found that the container also contained normal saline used for cleaning the suction tube, making it impossible to accurately calculate the sputum volume. To address this limitation, the most effective approach is to observe the suction process in real-time through video monitoring. Currently, our research team is planning to develop a method for real-time observation of the airway suction process, similar to bronchoscopy.

## Conclusions

This study examined the extent of airway mucosal injury, occurrence of ventilator-associated pneumonia, and adverse events to establish that the safety of the clinical trial medical device (LMECA.A1000) were not inferior to those of the conventional manual method.

## Data Availability

The datasets used and/or analyzed during the current study are available from the corresponding author upon reasonable request.

## Abbreviations

ABGA: Arterial blood gas analysis
ANC: Absolute neutrophil count
APACHE II: Acute Physiology and Chronic Health Evaluation II
CPR: Cardiopulmonary resuscitation
CRP: C-reactive protein
ICU: Intensive care unit
IQR: Interquartile range
PEEP: Positive end expiratory pressure
PT: Prothrombin time
SAPS III: Simplified Acute Physiology Score III
SD: Standard deviation
TEAE: Treatment-emergent adverse events
WBC: White blood cells
